# The impact of psychosocial factors on secondary hyperparathyroidism and vitamin D deficiency in adults with congenital heart disease—the CHD-HYPER study

**DOI:** 10.3389/fnut.2026.1681346

**Published:** 2026-04-27

**Authors:** Friederike Löffler, Kirsten Linhorst, Justus Christian Garlichs, Holger Leitolf, Christoph Terkamp, Ann-Sophie Silber-Peest, Johann Bauersachs, Kai G. Kahl, Mechthild Westhoff-Bleck

**Affiliations:** 1 Department of Cardiology and Angiology, Hannover Medical School, Hannover, Germany; 2 Department of Gastroenterology, Hepatology, Infectiology and Endocrinology, Hannover Medical School, Hannover, Germany; 3 Department of Psychiatry, Social Psychiatry and Psychotherapy, Hannover Medical School, Hannover, Germany

**Keywords:** 25-hydroxyvitamin D, adult congenital heart disease, depression, heart failure, hyperparathyroidism, parathyroid hormone, quality of life, vitamin D deficiency

## Abstract

**Background:**

Secondary hyperparathyroidism (sHPT) and vitamin D deficiency are common comorbidities in adults with congenital heart disease (ACHD), but the relationship with lifestyle factors, and psychosocial health remains poorly understood.

**Methods:**

This single-center cross-sectional study included 662 ACHD patients (mean age: 39.3 years; 47% female) and analyzed their clinical, psychosocial, and lifestyle data, including laboratory parameters, quality of life (QoL), and mental health metrics. Predictors of sHPT and vitamin D deficiency were evaluated using multivariate regression models.

**Findings:**

sHPT was observed in 13% and vitamin D deficiency in 43.4% of patients. In the multivariate analysis, infrequent alcohol consumption (*p* < 0.05), reduced QoL (*p* < 0.05), fewer headaches (*p* < 0.05), higher NYHA class (*p* < 0.05), elevated NT-proBNP levels (*p* < 0.05), metabolic syndrome (*p* < 0.05), and lower exercise frequency (*p* < 0.05) were independent predictors of sHPT. Reduced QoL (*p* < 0.05), less frequent alcohol consumption (*p* < 0.01), lower exercise frequency (*p* < 0.05) and shorter exercise duration (*p* < 0.01), as well as not living in a partnership (*p* < 0.01) were independent predictors of vitamin D deficiency.

**Conclusion:**

This study highlights the multifactorial etiology of sHPT and vitamin D deficiency in ACHD, underscoring the roles of disease severity, lifestyle, and psychosocial factors. sHPT appears to be closely associated with advanced cardiac disease and heart failure and may serve as a marker of increased disease burden. Accordingly, assessment of sHPT may contribute to a more comprehensive risk stratification in ACHD. Further studies are needed to determine whether targeted interventions improve clinical outcomes.

## Graphical abstract

**Figure:**
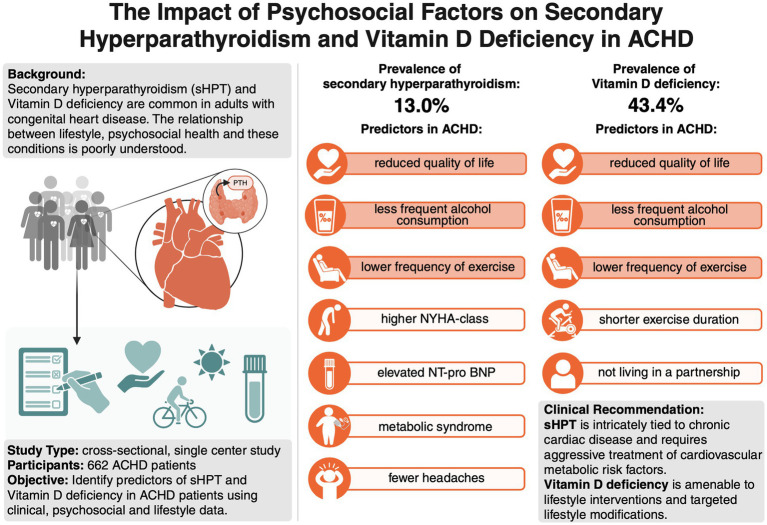

## Introduction

Congenital heart disease is the most common congenital disorder, affecting approximately 1% of live births (1). Due to major advances in pediatric cardiology and cardiac surgery, more than 90% of affected individuals now survive into adulthood (2). As a result, adult congenital heart disease (ACHD) has become a large and expanding population, with an estimated adult prevalence in the range of roughly 3 per 1,000 adults globally (3). ACHD can be classified according to the Bethesda classification into simple (e.g., isolated defects such as atrial septal defects or small ventricular septal defects), moderate (tetralogy of Fallot, atrioventricular septal defects, or coarctation of the aorta), and complex lesions (cyanotic congenital heart disease, single-ventricle physiology, Fontan circulation) (4). ACHD patients are not only at increased risk for cardiac complications, including heart failure and arrhythmias, but also for a wide range of extracardiac comorbidities, such as metabolic syndrome and chronic kidney disease (5, 6).

Secondary hyperparathyroidism (sHPT) is a significant comorbidity in adults with congenital heart disease (ACHD) and a recognized complication of heart failure (7–9). In heart failure, activation of the renin-angiotensin-aldosterone system (RAAS) induces hyperaldosteronism, leading to elevated parathyroid hormone (PTH) levels due to calcium loss through feces and kidneys. Loop diuretics, commonly used in heart failure treatment, further exacerbate sHPT by increasing renal calcium excretion (10). PTH plays a detrimental role in heart failure progression by stimulating aldosterone secretion via the adrenal PTH receptor and amplifying angiotensin II-induced aldosterone release (11). Furthermore, PTH increases intracellular calcium in cardiomyocytes, causing calcium overload, oxidative stress, myocardial cell death, and fibrosis (12, 13). In ACHD, vitamin D deficiency, impaired renal function, loop diuretics, low oxygen saturation, liver stiffness, and right ventricular dysfunction, are key predictors of sHPT (9). However, research into the interplay between psychosocial factors, sHPT, and vitamin D deficiency in ACHD is limited. Vitamin D deficiency is a widespread public health issue, with only 38.4% of German adults achieving adequate levels. Among the remainder, 30.2% are deficient, and 31.4% exhibit suboptimal levels (14). Vitamin D is essential for calcium and phosphorus homeostasis, enhancing their absorption in the gut, mobilizing these minerals from bone, and regulating renal calcium reabsorption and phosphorus excretion (15). Lifestyle factors such as low dietary calcium intake, high phosphorus consumption from processed foods, vitamin D-deficient diets, insufficient sunlight exposure, physical inactivity, and smoking contribute to vitamin D deficiency and sHPT (16–22). Obesity compounds these issues by sequestering vitamin D in adipose tissue (15). Mental health disorders, including depression and anxiety, may also influence sHPT and vitamin D deficiency. Depression has been associated with reduced vitamin D levels and sHPT in older adults (23) and with vitamin D deficiency in individuals with cardiovascular disease (24). Psychological comorbidities are particularly significant in ACHD, where they substantially affect quality of life (QoL) (25). Quality of life is often reduced in ACHD patients with more advanced disease, particularly in those with heart failure, cyanosis, or complex anatomical defects (26). Advances in medical and surgical care have significantly increased the life expectancy of individuals with congenital heart disease (27). The presence of vitamin D deficiency and sHPT in this population may worsen cardiovascular complications, impair bone health, and negatively impact QoL (28, 29). However, research in this area remains limited. This study aims to fill this gap by examining the impact of psychological comorbidities, QoL, and lifestyle factors on vitamin D deficiency and sHPT in ACHD.

## Materials and methods

### Study group

**Figure 1:**
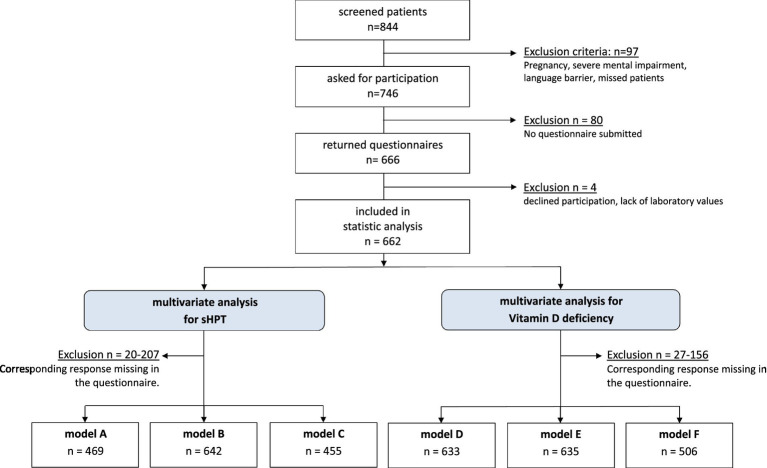
Patient inclusion tree.

This single-center cross-sectional study was conducted at Hannover Medical School and received approval from the institution’s ethics committee. All participants provided written informed consent prior to inclusion. Inclusion criteria included individuals aged over 18 years with a documented history of congenital heart disease. Exclusion criteria were pregnancy, malignancy, active systemic infection, withdrawal of consent, severe cognitive impairment, or significant language barriers that could interfere with study participation. Between August 2023 and January 2024, 844 patients were screened. Of these, 746 adult patients were invited to complete the questionnaire, and 666 returned it. Ultimately, 662 patients were included in the final analysis (Figure 1).

The questionnaire covered various aspects, such as educational background, dietary habits, physical activity, relationships, substance use, alcohol consumption, smoking habits, as well as pain assessment.

Health satisfaction and overall QoL were assessed using an analog scale with the following categories: very poor, poor, average, good, and excellent.

The UCLA 3-Item Loneliness Scale was used to assess levels of loneliness, with scores ranging from 3 to 9. A score of 3 indicates the lowest level of loneliness, while a score of 9 reflects the highest level of loneliness (30).

The Hospital Anxiety and Depression Scale (HADS) was used to assess anxiety (HADS-A), depression (HADS-D), and overall emotional distress (total HADS score). Scores of ≤7 on the HADS-A or HADS-D subscales indicate the absence of anxiety or depression, respectively (31).

Baseline clinical data, including initial cardiac diagnosis, Bethesda classification, cardiovascular surgeries and interventions, and cardiovascular complications (e.g., history of arrhythmias or cardiac decompensation), were collected from electronic medical records. Additional data obtained at the inclusion visit included NYHA classification, gender, age, height, weight, body mass index (BMI), pulse oximetry readings, laboratory results, current medications, and echocardiographic findings.

Given the diverse range of defects encompassed by congenital heart disease, patients were categorized into 14 specific groups: simple shunt defects, complex shunt defects, Ebstein anomaly, left ventricular outflow tract anomalies, aortic coarctation, conotruncal abnormalities, congenital pulmonary stenosis, Tetralogy of Fallot, pulmonary atresia with biventricular repair (with or without a ventricular septal defect), systemic right ventricle morphology (including d-transposition of the great arteries repaired via Mustard or Senning procedures, as well as congenitally corrected transposition), congenital aortopathies (e.g., Marfan syndrome, Loeys-Dietz syndrome, hereditary aortopathy), defects repaired with the Fontan procedure, Eisenmenger syndrome or precapillary pulmonary hypertension associated with congenital heart disease (PAH-CHD), including patients with functionally univentricular heart defects that are either unpalliated (native) or partially palliated, and various other heart defects not fitting into the previous categories (miscellaneous).

### Laboratory measurements

Serum concentrations of sodium, potassium, calcium, phosphate, and liver function markers (including alkaline phosphatase, gamma-glutamyltransferase, aspartate aminotransferase, alanine aminotransferase, cholinesterase, and bilirubin) were measured, along with kidney function (creatinine clearance), albumin, thyroid-stimulating hormone, HbA1c, lipid profile, and a complete blood count, using standard laboratory methods. Systemic inflammation markers, such as CRP and growth differentiation factor 15 (GDF-15), as well as N-terminal pro-B-type natriuretic peptide (NT-proBNP) levels, were also evaluated.

To assess vitamin D status and sHPT, levels of 25-hydroxyvitamin D and intact PTH were measured with standard laboratory techniques. According to the Endocrine Society’s Practice Guidelines on Vitamin D, deficiency was defined as 25-hydroxyvitamin D levels below 20 ng/mL, while insufficiency was defined as 21–29 ng/mL (32).

SHPT was diagnosed when intact PTH levels exceeded 65 pg./mL while serum calcium levels remained within the normal range, as per the cut-off set by the institutional biochemistry lab. No cases of primary hyperparathyroidism, characterized by both elevated serum calcium and PTH, were found in this study. Metabolic syndrome was defined as the presence of at least three of the following criteria: waist size ≥ 88 cm (female) or ≥102 cm (male), high density lipoproteins < 50 mg/dL (female) or <40 mg/dL (male), blood pressure ≥ 130 mmHg systolic or ≥ 85 mmHg diastolic, HbA1c ≥ 5.7%).

### Statistical analysis

Data were analyzed using IBM SPSS Statistics version 29.0.1.0 (SPSS Inc., Troy, NY). Differences between groups in continuous and categorical variables were calculated using unpaired t-test, Mann–Whitney-U test or the χ2 test as appropriate. Two-sided tests were used throughout. A value of *p* < 0.05 was considered to be significant.

We employed multivariate binary logistic regression to identify predictors associated with the presence of sHPT. Initially, a univariate analysis was performed, after which significant variables from this analysis were incorporated into a multivariate regression model, tailored according to the selected approach. Only participants with available data for the relevant variables were included in the analysis.

## Results

### Study population

Baseline demographics, clinical characteristics, and laboratory data are presented in Table 1. The study included a total of 662 patients, with a median age of 39.3 years at inclusion, and 351 participants (53%) were male. Based on the NYHA classification, 487 patients (73.6%) were in class I, 129 (19.5%) in class II, and 46 (6.9%) were in classes III and IV.

**Table 1:** Comparison between patients with sHPT and patients without sHPT and patients with Vitamin D deficiency and without Vitamin D deficiency.

	Total cohort		Secondary hyperparathyroidism			Vitamin D		
	n	mean ± SD	sHPT* (*n* = 86) n(%) or mean ± SD	No sHPT* (*n* = 576) n(%) or mean ± SD	*p* value	Vit. D deficiency^+^ (*n* = 287) n(%) or mean ± SD	No Vit. D deficiency^+^ (*n* = 375) n(%) or mean ± SD	*p* value
Age (years)	662	39.3 ± 12.6	43.8 ± 14.3	38.6 ± 12.2	**<0.001**	37.5 ± 12.6	40.7 ± 12.8	**0.001**
Male	662	351 (53.0)	43 (50.0)	308 (53.5)	0.547	163 (56.8)	188 (50.1)	0.089
^1^School education (years)	658	11.5 ± 1.2	11.3 ± 1.2	11.5 ± 1.1	0.089	11.5 ± 1.1	11.5 ± 1.2	0.633
Vocational training	660	557 (84.1)	71 (82.6)	486 (84.7)	0.615	229 (80.4)	328 (87.5)	**0.013**
^2^BMI (kg/m^2^)	662	25.9 ± 5.3	26.8 ± 6.2	25.7 ± 5.1	0.126	26.3 ± 5.7	25.6 ± 4.9	0.117
^3^Metabolic syndrome	662	77 (11.6)	19 (22.1)	58 (10.1)	**0.001**	41 (14.2)	36 (9.6)	0.062
Arterial hypertonia	660	172 (26.0)	30 (34.9)	142 (24.7)	**0.046**	66 (23.1)	106 (28.3)	0.127
Diabetes mellitus	657	27 (4.1)	7 (8.1)	20 (3.5)	**0.040**	14 (4.9)	13 (3.5)	0.359
^4^Bethesda classification	662							
Simple		110 (16.6)	13 (15.1)	97 (16.8)		40 (13.9)	70 (18.7)	
Moderate		174 (26.3)	12 (14.0)	162 (28.1)		79 (27.5)	95 (25.3)	
Complex		378 (57.1)	61 (70.9)	317 (55.0)		168 (58.5)	210 (56.0)	
Mean		1.6 ± 0.8	1.4 ± 0.7	1.6 ± 0.8	**0.018**	1.6 ± 0.7	1.6 ± 0.8	0.304
^5^NYHA	662							
Class I		487 (73.6)	42 (48.8)	445 (77.3)		200 (69.7)	287 (76.5)	
Class II		129 (19.5)	30 (34.9)	99 (17.2)		62 (21.6)	67 (17.9)	
Classes III + IV		46 (6.9)	14 (16.3)	32 (5.6)		25 (8.7)	21 (5.6)	
Mean		1.3 ± 0.6	1.7 ± 0.7	1.3 ± 0.6	**<0.001**	1.4 ± 0.7	1.3 ± 0.6	**0.039**
^6^NT-proBNP (ng/l)	661	306.3 ± 1154.8	640.6 ± 1463.7	256.4 ± 1095.0	**0.004**	272.8 ± 814.5	332.0 ± 1360.1	0.487
25-Hydroxyvitamin D (ng/ml)	661	22.8 ± 10.5	20.6 ± 9.2	23.1 ± 10.7	**0.039**	14.7 ± 3.6	29.0 ± 9.9)	**<0.001**
Vitamin D level	662							
Vitamin D deficiency (<20 ng/ml)		287 (43.4)	47 (54.7)	240 (41.7)				
Insufficient supply (20–29.9 ng/ml)		262 (39.6)	26 (30.2)	236 (41.0)				
Sufficient supply (>30 ng/mL)		113 (17.1)	13 (15.1)	100 (17.4)	0.051			
Vitamin D substitution	653	127 (19.4)	18 (20.9)	109 (19.2)	0.710	19 (6.6)	108 (29.5)	**<0.001**
Intact parathyroid hormone (pg/ml)	660	45.6 ± 40.4	96.4 ± 93.1	38.0 ± 12.1	**<0.001**	50.2 ± 55.7	42.0 ± 21.9	**0.009**
Parathyroid hormone elevated n(%)	662	86 (13.0)				47 (16.4)	39 (10.4)	**0.023**
^7^GFR (ml/min)	662	95.1 ± 19.5	83.7 ± 24.5	96.8 ± 18.1	**<0.001**	98.0 ± 18.0	93.0 ± 20.5	**<0.001**
aspartate aminotransferase (AST)	662	26.6 ± 11.3	29.6 ± 15.1	26.1 ± 10.6	**0.009**	27.2 ± 13.2	26.2 ± 9.7	0.266
Gamma-glutamyl transferase (gGT)	662	40.8 ± 52.3	59.5 ± 73.1	38.0 ± 48.0	**<0.001**	44.9 ± 60.9	37.8 ± 44.6	0.098
Transferrin saturation (*n* = 661)	661	28.9 ± 16.6	25.3 ± 11.4	29.5 ± 17.2	**0.031**	28.1 ± 12.3	29.6 ± 19.3	0.241
O_2_- saturation (*n* = 635)	635	97.2 ± 2.8	96.2 ± 3.8	97.4 ± 2.6	**<0.001**	97.2 ± 2.8	97.3 ± 2.9	0.713
Physical activity	657	574 (86.7)	67 (79.8)	507 (88.5)	**0.025**	238 (83.5)	336 (90.3)	**0.009**
Frequency of exercise	655							
Never		115 (17.6)	24 (28.2)	91 (16.0)		65 (22.7)	50 (13.6)	
Monthly		184 (28.1)	27 (31.8)	157 (27.5)		83 (29.0)	101 (27.4)	
Weekly		299 (45.6)	30 (35.3)	269 (47.2)		117 (40.9)	182 (49.3)	
Daily		57 (8.7)	4 (4.7)	53 (9.3)	**0.001**	21 (7.3)	36 (9.8)	**0.002**
Exercise duration (Minutes/Week)	516	300.2 ± 290.9	231.0 ± 249.0	309.3 ± 295.3	**0.028**	255.1 ± 251.7	334.0 ± 313.8	**0.002**
Time spent outdoors (Hours/Day)	657	2.9 ± 1.8	2.7 ± 1.7	2.9 ± 1.9	0.202	2.7 ± 1.7	3.0 ± 1.9	0.068
Dietary style	662							
Omnivorous		598 (90.3)	78 (90.7)	520 (90.3)	0.179	267 (93.0)	331 (88.3)	**0.040**
Vegetarian, vegan, other		64 (9.7)	8 (9.3)	56 (9.7)	0.179	20 (7.0)	44 (11.7)	**0.040**
Fish consumption	662	552 (83.4)	72 (83.7)	480 (83.3)	0.928	234 (81.5)	318 (84.8)	0.263
Meat consumption	662	607 (91.7)	80 (93.0)	527 (91.5)	0.632	271 (94.4)	336 (89.6)	**0.026**
Frequency of fast food consumption	660							
Never		95 (14.4)	18 (20.9)	77 (13.4)		31 (10.8)	64 (17.2)	
Monthly		488 (73.9)	57 (66.3)	431 (75.1)		214 (74.6)	274 (73.5)	
Weekly		74 (11.2)	9 (10.5)	65 (11.3)		41 (14.3)	33 (8.8)	
Daily		3 (0.5)	2 (2.3)	1 (0.2)	0.303	1 (0.3)	2 (0.5)	**0.004**
Alcohol consumption	659	400 (60.4)	37 (44.0)	363 (63.1)	**0.001**	156 (54.5)	244 (65.4)	**0.005**
Number of drinks per week	646	1.8 ± 3.8	1.2 ± 2.8	1.9 ± 3.9	0.050	1.9 ± 4.5	1.7 ± 3.1	0.633
Smoking	661	76 (11.5)	9 (10.5)	67 (11.7)	0.748	35 (12.2)	41 (11.0)	0.622
Drug use	660	22 (3.3)	1 (1.2)	21 (3.7)	0.235	11 (3.8)	11 (2.9)	0.531
Living in a partnership	662	202 (30.5)	30 (34.9)	172 (29.9)	0.345	176 (61.3)	284 (75.7)	**<0.001**
Children	660	295 (44.6)	43 (50.0)	252 (43.9)	0.289	109 (38.0)	186 (49.9)	**0.002**
^8^Health satisfaction (mean)	659	3.4 ± 0.9	3.2 ± 1.0	3.5 ± 0.9	**0.020**	3.4 ± 0.9	3.5 ± 0.9	0.272
^8^Quality of life (mean)	656	3.9 ± 0.7	3.7 ± 0.7	4.0 ± 0.7	**0.001**	3.8 ± 0.7	4.0 ± 0.7	**0.026**
^9^HADS-A	659	5.7 ± 3.9	6.0 ± 3.8	5.7 ± 3.9	0.534	5.6 ± 3.9	5.8 ± 3.9	0.525
^9^HADS-D	658	4.0 ± 3.5	4.4 ± 3.9	3.9 ± 3.4	0.269	4.1 ± 3.6	3.9 ± 3.4	0.471
^9^Total HADS score	659	9.7 ± 6.7	10.4 ± 7.0	9.6 ± 6.7	0.337	9.7 ± 6.3	9.7 ± 6.7	0.979
Headache	482	212 (44.0)	20 (29.9)	192 (46.3)	**0.012**	92 (45.1)	120 (43.2)	0.673

*Hyperparathyroidism was defined as a parathyroid hormone level ≥ 65 pg/ml, ^+^Vitamin D deficiency was defined as a 25-Hydroxyvitamin D level <20 ng/ml.

1 Years of Schooling.

2 Body mass index.

3 Defined as the presence of at least three of the following criteria: waist size ≥ 88 cm (female) or ≥102 cm (male), high density lipoproteins < 50 mg/dl (female) or <40 mg/dl (male), blood pressure ≥ 130 mmHg systolic or ≥ 85 mmHg diastolic, HbA1c ≥ 5.7%.

4 Complexity of heart defect according to AHA/ACC.

5 New York Heart Association Classification.

6 N-terminal pro brain natriuretic peptide.

7 Glomerular Filtration Rate.

8 Quality of life and health satisfaction were assessed using a rating scale from 1 to 5.

9 Hospital Anxiety and Depression Scale.

Bold numbers indicate statistically significant values.

According to the Bethesda classification, the cohort comprised 378 patients (57.1%) with complex congenital heart defects, 174 patients (26.3%) with moderate defects, and 110 patients (16.6%) with simple defects.

**Figure 2:**
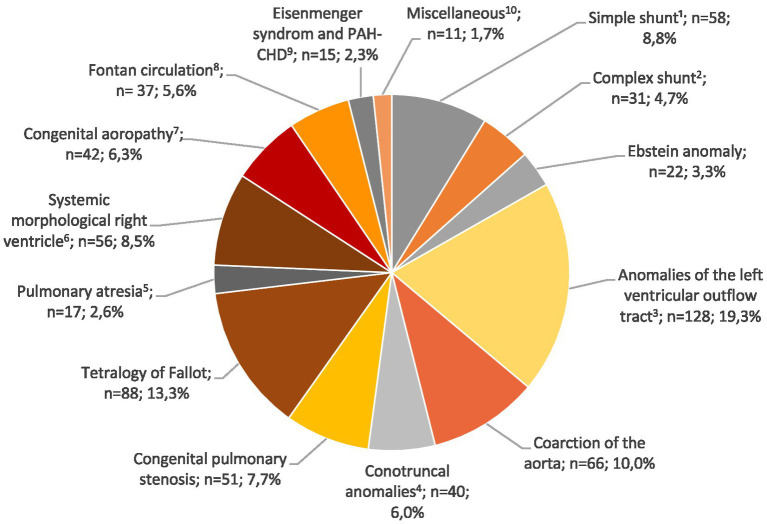
Pie chart depicting distribution of congenital heart defects. ^1^Simple shunt: hemodynamically irrelevant atrial septal defect, patent foramen ovale, ventricular septal defect; ^2^Complex shunt: abnormal pulmonary venous return, atrioventricular septal defect; ^3^Anomalies of the left ventricular outflow tract: mitral valve diseases, aortic anomalies; ^4^Conotruncal anomalies: biventricular repair of double outlet right ventricle and common arterial trunc, D-transposition of the great arteries after arterial-switch procedure; ^5^Pulmonary atresia with biventricular repair; ^6^Systemic morphological right ventricle: D-transposition of great arteries corrected through Mustard or Senning procedure, congenitally corrected transposition of the great arteries; ^7^Congenital aortopathy: comprising Marfan syndrome, Loeys-Dietz-syndrome, Ehlers-Danlos-syndrome, other forms of hereditary aortopathy; ^8^Fontan circulation; ^9^Eisenmenger syndrome/PAH-CHD: pulmonary arterial hypertension related to congenital heart disease according to Nizza 1.4; ^10^Miscellaneous: congenital heart defects that could not be assigned to the other groups.

The distribution of congenital heart defects is illustrated in Figure 2. The largest subgroup included anomalies of the left ventricular outflow tract (*n* = 128, 19.3%), followed by tetralogy of Fallot (*n* = 88, 13.3%), aortic coarctation (*n* = 66, 10%), simple shunts (*n* = 58, 8.8%), and systemic morphological right ventricle defects (*n* = 56, 8.5%).

SHPT was observed in 13% of the total patient cohort. 43.4% of all patients exhibited vitamin D deficiency, while 39.6% had insufficient levels of vitamin D. 19.4% of the patients received vitamin D supplementation.

Among patients with sHPT, 54.65% (*n* = 47) had a vitamin D deficiency. Conversely, among those with vitamin D deficiency, 16.38% exhibited sHPT (*p* = 0.027).

### Comparison between patient groups

A direct comparison of patients with sHPT revealed significant differences in age, with the sHPT group averaging 43.8 years compared to 38.6 years in the non-sHPT group (*p* < 0.001). Additionally, patients with sHPT were more likely to have complex congenital heart disease as classified by the Bethesda system (*p* = 0.018). They also exhibited worse NYHA functional class and higher NT-proBNP levels compared to patients without sHPT (all *p* < 0.004), indicating greater disease severity.

Further, various organ systems showed more pronounced functional impairment in the sHPT group. Glomerular filtration rate (GFR) was significantly lower, vitamin D levels were reduced, liver function test results were elevated, and both oxygen saturation and transferrin saturation were significantly lower (all *p* < 0.039). Moreover, metabolic syndrome was more often present in the sHPT group (*p* < 0.001).

Regarding lifestyle factors, patients with sHPT were less physically active, and their exercise duration was significantly shorter. However, dietary habits did not differ between the two groups (all *p* > 0.28). Notably, alcohol consumption was more frequent among patients without sHPT (*p* < 0.01).

In terms of subjective outcomes, the sHPT group reported lower health satisfaction and a reduced QoL compared to the non-sHPT group (all *p* < 0.02). There was no significant difference between the two groups regarding loneliness as measured by the UCLA Loneliness Scale, or in anxiety and depression scores assessed by the HADS (HADS-A, HADS-D, and total scores). Finally, headaches were reported more frequently by patients without sHPT (*p* < 0.012), see Table 1.

A direct comparison between patients with and without vitamin D deficiency revealed significant differences in age; patients without vitamin D deficiency were older (*p* < 0.001). Unlike the earlier comparison between patients with sHPT and those without, no differences were found in terms of Bethesda class, NT-proBNP levels, or metabolic syndrome between the two groups.

Patients with vitamin D deficiency reported a lower frequency of exercise and were less likely to follow a vegetarian diet (all *p* < 0.04). Conversely, patients without vitamin D deficiency consumed alcohol more frequently (*p* = 0.005). Patients without vitamin D deficiency were also more likely to live in a partnership and have children (both *p* < 0.002).

The mean HADS-A score for the entire population was 5.7 ± 3.9, the mean HADS-D score was 4.0 ± 3.5, and the mean total HADS score was 9.7 ± 6.7, indicating and absence of depression or anxiety (33). Comparing patient with vitamin D deficiency and those without, no differences were observed for HADS-A, HADS-D, total HADS scores, and UCLA Loneliness Scale (all *p* > 0.629). Similarly, the frequency of headaches did not differ between the groups (*p* = 0.673). Not surprisingly, GFR, PTH levels, and the rate of vitamin D supplementation were significantly different (*p* < 0.023), see Table 1.

Given the significant differences in alcohol consumption between patients with sHPT or vitamin D deficiency and those without, the patients were divided into two groups based on their alcohol intake. The first group included individuals consuming 1.8 or fewer drinks per week, while the second group consisted of those consuming more than 1.8 drinks per week, which was the average weekly alcohol consumption. Patients in the higher alcohol consumption group exhibited a significantly lower mean UCLA isolation score (*p* = 0.025; CI = 0.033–0.483), better quality of life [*p* = 0.013; CI = −0.271–(−0.031)], greater health satisfaction [*p* = 0.009; CI = −0.3562–(−0.0488)], and a lower functional NYHA class (*p* < 0.001, CI = 0.094–0.269), see Table 2.

**Table 2:** Comparison of the study population in alcohol consumption.

	n	Total cohort mean ± SD	≤1.8 drinks per week (*n* = 449) mean ± SD	>1.8 drinks per week (*n* = 197) mean ± SD	*p* value
^1^Loneliness scale	657	3.9 ± 1.4	4.1 ± 1.5	3.75 ± 1.2	**0.025**
^2^Bethesda classification	662	1.6 ± 0.8	1.61 ± 0.8	1.57 ± 0.7	0.520
^3^NYHA class	662	1.34 ± 0.6	1.39 ± 0.7	1.2 ± 0.5	**<0.001**
^4^Frequency of exercise	655	2.45 ± 0.9	2.44 ± 0.9	2.54 ± 0.8	0.176
^5^QoL	656	3.91 ± 0.7	3.87 ± 0.8	4.03 ± 0.7	**0.013**
^6^Health satisfaction	659	3.42 ± 0.9	3.32 ± 0.9	3.6 ± 0.9	**0.009**

1 Three-Item Loneliness Scale.

2 Complexity of heart defect according to AHA/ACC.

3 New York Heart Association Classification.

4 Frequency of Exercise was assessed using a rating scale from 1 to 4 (never, monthly, weekly, daily).

5 Quality of life was assessed using a rating scale from 1 to 5.

6 Health Satisfaction was assessed using a rating scale from 1 to 5.

Bold numbers indicate statistically significant values.

A comparison between patients taking vitamin D supplements and those not taking them revealed no significant differences in NYHA functional class, Bethesda class, or NT-proBNP levels (all *p* > 0.067).

### Univariate and multivariate analyses

Results from univariate analyses for sHPT and vitamin D deficiency are shown in Table 3.

**Table 3:** Univariate regression analysis.

*n* = 662	sHPT*				Vitamin D Deficiency^+^			
	*OR*	95% CI		*p* value	*OR*	95% CI		*p* value
		*LL*	*UL*			*LL*	*UL*	
Age	1.030	1.013	1.048	**<0.001**	0.980	0.967	0.992	**<0.001**
Male	0.870	0.553	1.369	0.548	1.308	0.960	1.781	0.089
^1^School education (*n* = 658)	0.717	0.504	1.020	0.064	1.066	0.829	1.370	0.618
Vocational training (*n* = 660)	0.857	0.470	1.564	0.615	0.586	0.384	0.894	**0.013**
Living in a Partnership	0.795	0.493	1.282	0.346	0.508	0.636	0.710	**<000.1**
^2^Metabolic syndrome	2.533	1.422	4.511	**0.002**	1.569	0.974	2.528	0.064
Arterial hypertonia (*n* = 660)	1.630	1.006	2.640	**0.047**	0.758	0.532	1.082	0.127
^3^Bethesda classification	1.400	1.006	1.947	**0.046**	1.136	0.926	1.394	0.222
^4^NYHA	2.365	1.725	3.243	**<0.001**	1.314	1.019	1.696	**0.036**
^5^NT-proBNP (*n* = 661)	1.000	1.000	1.000	0.066	1.000	1.000	1.000	0.530
25-Hydroxyvitamin D (*n* = 661)	0.973	0.948	0.999	**0.043**				
^+^Vitamin D deficiency (<20 ng/ml)	1.687	1.070	2.661	**0.024**				
Vitamin D substitution (*n* = 653)	1.112	0.635	1.947	0.710	0.169	0.101	0.284	**<000.1**
*sHPT (PTH level ≥ 65 pg./ml)					1.687	1.070	2.661	**0.024**
^6^GFR	0.969	0.958	0.980	**<0.001**	1.014	1.006	1.022	**<000.1**
Aspartate Aminotransferase (AST)	1.019	1.003	1.036	**0.018**	1.008	0.994	1.022	0.255
Gamma-Glutamyl Transferase (gGT)	1.005	1.002	1.009	**0.002**	1.003	1.000	1.006	0.093
Transferrin Saturation (*n* = 661)	0.971	0.950	0.992	**0.007**	0.994	0.983	1.005	0.284
O_2_- Saturation (*n* = 635)	0.893	0.838	0.952	**<0.001**	0.990	0.937	1.046	0.714
Physical activity (*n* = 657)	0.513	0.284	0.926	**0.027**	0.543	0.341	0.864	**0.010**
^7^Frequency of exercise (*n* = 655)	0.653	0.504	0.846	**<0.001**	0.747	0.625	0.892	**<0.001**
^8^Exercise duration (*n* = 516)	0.999	0.997	1.000	**0.047**	0.999	0.998	1.000	**0.003**
^9^Time outdoors (*n* = 657)	0.920	0.804	1.053	0.226	0.924	0.848	1.008	0.074
^10^Fast food consumption (*n* = 660)	0.861	0.557	1.331	0.501	1.527	1.130	2.064	**0.006**
Alcohol consumption (*n* = 659)	0.460	0.289	0.730	**<0.001**	0.634	0.463	0.870	**0.005**
^11^Number of drinks (*n* = 646)	0.922	0.832	1.022	0.121	1.011	0.970	1.053	0.616
Smoking (*n* = 661)	0.886	0.425	1.850	0.748	1.128	0.698	1.823	0.623
Drug use (*n* = 660)	0.314	0.042	2.366	0.261	1.312	0.560	3.070	0.532
^12^HADS-D (*n* = 659)	1.039	0.977	1.105	0.226	1.016	0.973	1.062	0.468
^12^HADS-A (*n* = 658)	1.018	0.961	1.079	0.534	0.987	0.948	1.027	0.525
^12^Total HADS score (*n* = 659)	1.017	0.984	1.051	0.316	1.000	0.977	1.023	0.979
^13^Health Satisfaction (*n* = 659)	0.750	0.589	0.955	**0.019**	0.905	0.766	1.069	0.242
^13^Quality of life (*n* = 656)	0.604	0.448	0.813	**<0.001**	0.789	0.640	0.974	**0.027**
Headache (*n* = 482)	0.494	0.283	0.863	**0.013**	1.082	0.752	1.556	0.673

*Secondary hyperparathyroidism was defined as parathyroid hormone level ≥ 65 pg/ml, ^+^Vitamin D deficiency was defined as a 25-Hydroxyvitamin D level <20 ng/ml.

1 Years of Schooling.

2 defined as the presence of at least three of the following criteria: waist size ≥ 88 cm (female) or ≥102 cm (male), high density lipoproteins < 50 mg/dl (female) or <40 mg/dl (male), blood pressure ≥ 130 mmHg systolic or ≥ 85 mmHg diastolic, HbA1c ≥ 5.7%.

3 Complexity of heart defect according to AHA/ACC.

4 New York Heart Association classification.

5 N-terminal pro brain natriuretic peptide.

6 Glomerular Filtration Rate.

7 Frequency of Exercise was assessed using a rating scale from 1 to 4 (never, monthly, weekly, daily).

8 Exercise duration in minutes per week.

9 Time outdoors in hours per day.

10 Frequency of fast-food consumption was assessed using a rating scale from 1 to 4 (never, monthly, weekly, daily).

11 Number of Drinks per week.

12 Hospital Anxiety and Depression Scale.

13 Quality of life and health satisfaction were assessed using a rating scale from 1 to 5.

Bold numbers indicate statistically significant values.

We developed multiple multivariate regression models to assess the impact of psychosocial and lifestyle factors as well as physical factors on the development of sHPT and vitamin D deficiency. Variables included in the multivariate analyses were selected based on results from univariate analyses. Predominantly, significant factors identified in the univariate analyses were incorporated, alongside variables deemed relevant to the pathogenesis of sHPT and vitamin D deficiency.

### Models for sHPT

All models assessing sHPT were adjusted for age, sex, GFR, and vitamin D deficiency. After adjustment, vitamin D deficiency and reduced GFR remained independent predictors of sHPT across all models, underscoring their distinct associations with the condition.

#### Model A

Model A included metabolic syndrome, NYHA class, Bethesda classification, alcohol consumption, QoL, health satisfaction, frequency of exercise, and the presence of headaches. Additionally, school education, reflecting socioeconomic status, was incorporated despite lacking statistical significance in univariate analysis.

**Figure 3:**
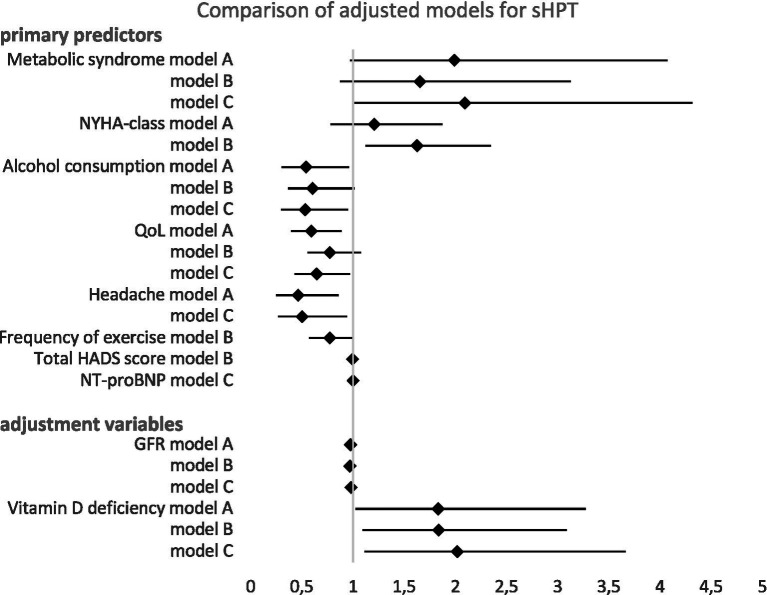
Forest plot showing odds ratios with 95% confidence intervals for the association with sHPT in multivariate analysis models A–C. The diamond represents the odds ratio. Values greater than 1.0 indicate increased risk, while values less than 1.0 indicate decreased risk. Models A–C were adjusted for age, sex, GFR, and vitamin D deficiency. Detailed model specifications in the Supplementary part Table A1.

After adjusting for age, sex, GFR, and vitamin D deficiency, less frequent alcohol consumption, reduced QoL, and fewer headaches emerged as significant predictors for sHPT in ACHD. GFR and vitamin D deficiency remained significant predictors following these adjustments (Table 4; Figure 3).

**Table 4:** Multivariate analysis results for sHPT.

Variables	Model A, unadjusted OR (95% CI)	Model A OR (95% CI)	Model B OR (95% CI)	Model C OR (95% CI)
^1^Metabolic syndrome	2.175 (1.080–4.380)*	1.989 (0.972–4.070)	1.653 (0.874–3.126)	2.091 (1.013–4.315)*
^2^NYHA-class	1.530 (1.027–2.277)*	1.209 (0.781–1.872)	1.623 (1.124–2.345)*	---
Alcohol consumption	0.534 (0.304–0.937)*	0.541 (0.304–0.961)*	0.609 (0.366–1.014)	0.531 (0.296–0.954)*
^3^QoL	0.569 (0.386–0.839)**	0.594 (0.398–0.886)*	0.775 (0.557–1.078)	0.646 (0.430–0.969)*
^3^Health satisfaction				---
Headache	0.392 (0.216–0.712)**	0.464 (0.252–0.857)*	---	0.503 (0.269–0.941)*
^4^Frequency of exercise	0.810 (0.596–1.102)	---	0.775 (0.573–0.995)*	---
^5^Total HADS score	---	---	0.995 (0.955–1.037)	---
^6^NT-proBNP	---	---	---	1.001 (1.000–1.001)*
^7^GFR	---	0.976 (0.961–0.992)**	0.973 (0.961–0.986)***	0.980 (0.964–0.997)*
^8^Vitamin D deficiency	---	1.832 (1.025–3.272)*	1.838 (1.094–3.085)*	2.019 (1.113–3.661)*

*p < 0.05, **p < 0.01, ***p < 0.001. Multivariate odds ratios were calculated to assess associations with sHPT. Hyperparathyroidism was defined as a parathyroid hormone level ≥ 65 pg/ml. Models A-C were adjusted for age, sex, GFR and Vitamin D deficiency. All models include patients who provided corresponding response in the questionnaire. Detailed model specifications in the Supplementary part Table A1.

1 defined as the presence of at least three of the following criteria: waist size ≥ 88 cm (female) or ≥102 cm (male), high density lipoproteins < 50 mg/dl (female) or <40 mg/dl (male), blood pressure ≥ 130 mmHg systolic or ≥ 85 mmHg diastolic, HbA1c ≥ 5.7%.

2 New York Heart Association Classification.

3 Quality of life and health satisfaction were assessed using a rating scale from 1 to 5.

4 Frequency of Exercise was assessed using a rating scale from 1 to 4 (never, monthly, weekly, daily).

5 Hospital Anxiety and Depression Scale.

6 N-terminal pro brain natriuretic peptide.

7 Glomerular Filtration Rate.

8 Vitamin D deficiency was defined as a 25-Hydroxyvitamin D level <20 ng/ml.

#### Model B

Model B examined the same variables as Model A, with the exception of headache, which was replaced by the total HADS (Hospital Anxiety and Depression Scale) score to evaluate the influence of depression and anxiety on sHPT. After adjustment for age, sex, GFR, and vitamin D deficiency, higher NYHA class and less frequent exercise were identified as independent predictors of sHPT. GFR and vitamin D deficiency remained significant predictors following these adjustments (Table 4; Figure 3).

#### Model C

Model C investigated the influence of metabolic syndrome, alcohol consumption, QoL, and headache, along with NT-proBNP, transferrin saturation, oxygen saturation, and liver function (represented by γGT), on sHPT. After adjustment for age, sex, GFR, and vitamin D deficiency, less frequent alcohol consumption, reduced QoL, and fewer headaches remained significant predictors. Furthermore, the presence metabolic syndrome and elevated NT-proBNP levels also attained statistical significance in this model. GFR and vitamin D deficiency remained significant predictors following these adjustments (Table 4; Figure 3).

### Models for vitamin D deficiency

Three additional models (Models D, E, F) explored factors independently associated with vitamin D deficiency.

The following models were adjusted for age, sex, GFR, and vitamin D substitution. After adjustment, absence of vitamin D supplementation and lower GFR were consistently identified as independent predictors of vitamin D deficiency in all models, emphasizing their strong association with the condition.

#### Model D

This model examined metabolic syndrome, NYHA class, Bethesda class, alcohol consumption frequency, QoL, health satisfaction, exercise frequency, total HADS score, and fast-food consumption frequency. NT-proBNP was not included in this model, as it did not reach significance in univariate analyses (*p* = 0.53), see Table 3. After adjustment, reduced QoL and less frequent alcohol consumption emerged as significant predictors of vitamin D deficiency. Metabolic syndrome, NYHA class, Bethesda classification, fast food consumption, frequency of exercise, and total HADS score were not significant predictors.

**Figure 4:**
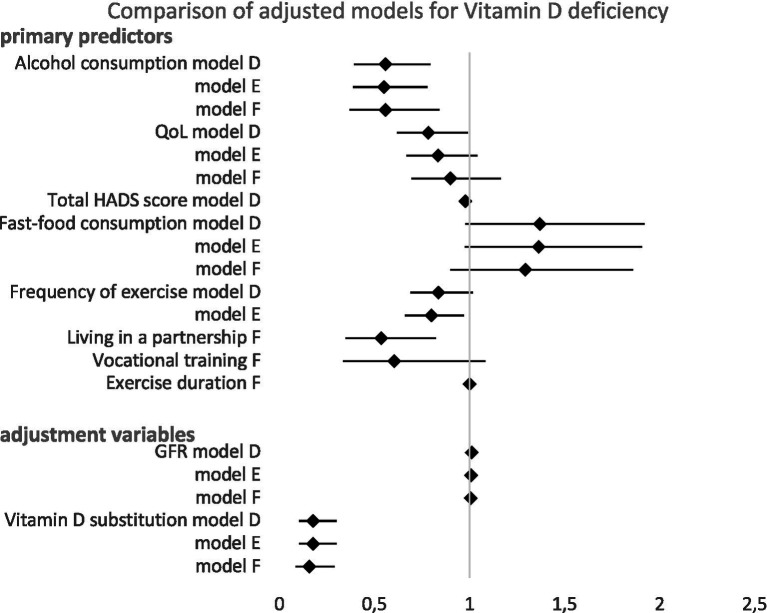
Forest plot showing odds ratios with 95% confidence intervals for the association with vitamin D deficiency in multivariate analysis models (D–F). The diamond represents the odds ratio. Values greater than 1.0 indicate increased risk, while values less than 1.0 indicate decreased risk. Models (D–F) were adjusted for age, sex, GFR, and vitamin D substitution. Detailed model specifications in the Supplementary part Table A2.

However, after adjustment, fast food consumption and frequency of exercise narrowly missed statistical significance. Vitamin D substitution and GFR remained significant predictors after the aforementioned adjustments (all *p* < 0.017), see Table 5 and Figure 4.

**Table 5:** Multivariate analysis results for Vitamin D deficiency.

Variables	Model D, unadjusted OR (95% CI)	Model D OR (95% CI)	Model E OR (95% CI)	Model F OR (95% CI)
Alcohol consumption	0.638 (0.458–0.887)**	0.559 (0.393–0.794)**	0.549 (0.388–0.778)***	0.558 (0.370–0.841)**
^1^QoL	0.882 (0.658–1.026)	0.784 (0.619–0.992)*	0.833 (0.668–1.040)	0.899 (0.695–1.164)
^2^Total HADS score	0.979 (0.952–1.008)	0.979 (0.952–1.008)	---	---
^3^Fast-food consumption	1.506 (1.095–2.072)*	1.370 (0.978–1.919)	1.363 (0.974–1.908)	1.293 (0.899–1.860)
^3^Frequency of exercise	0.817 (0.677–0.985)*	0.838 (0.689–1.018)	0.800 (0.660–0.970)*	---
Living in a partnership	---	---	---	0.534 (0.347–0.822)**
Vocational training	---	---	---	0.602 (0.335–1.084)
^4^Exercise duration	---	---	---	0.999 (0.998–1.000)**
^5^GFR	---	1.011 (1.002–1.021)*	1.010 (1.001–1.020)*	1.006 (0.993–1.020)
Vitamin D substitution	---	0.176 (0.103–0.301)***	0.176 (0.103–0.300)***	0.158 (0.086–0.291)***

*p < 0.05, **p < 0.01, ***p < 0.001. Multivariate odds ratios were calculated to assess associations with Vitamin D deficiency. Vitamin D deficiency was defined as a 25-Hydroxyvitamin D level <20 ng/ml. Models D-F were adjusted for age, sex, GFR and Vitamin D substitution. All models include patients who provided corresponding response in the questionnaire. Detailed model specifications in the Supplementary part Table A2.

1 Quality of life was assessed using a rating scale from 1 to 5.

2 Hospital Anxiety and Depression Scale.

3 Frequency of fast-food consumption and frequency of exercise were assessed using a rating scale from 1 to 4 (never, monthly, weekly, daily).

4 Exercise duration was asked in minutes per week.

5 Glomerular Filtration Rate.

#### Model E

This model included the same variables as Model D, with exception of the total HADS score and health satisfaction, while incorporating γGT and transferrin saturation to assess physical contributors to vitamin D deficiency.

After adjusting for age, sex, GFR, and vitamin D supplementation, less frequent exercise and lower alcohol consumption were identified as significant predictors. Vitamin D substitution and GFR remained significant predictors even after the aforementioned adjustments (all *p* < 0.026), see Table 5 and Figure 4.

#### Model F

After adjusting for age, sex, GFR, and vitamin D supplementation, less frequent alcohol consumption (*p* = 0.005), shorter duration of exercise (*p* = 0.005) and not living in a partnership (*p* = 0.004) emerged as significant predictors of vitamin D deficiency. In this model, neither NYHA functional class, Bethesda class, active smoking, having children, QoL, nor fast food consumption were significant predictors of vitamin D deficiency. Vitamin D substitution remained a significant predictor following these adjustments (*p* < 0.001), see Table 5 and Figure 4.

## Discussion

To, date, our study is the first to provide a comprehensive analysis of psychosocial and lifestyle factors associated with sHPT and vitamin D deficiency in ACHD. Our findings highlight the complex interplay between physical health, lifestyle, and psychosocial factors in the development of these conditions.

### Secondary hyperparathyroidism

Previous studies have demonstrated that sHPT is more prevalent in patients with complex and advanced CHD compared with those with less severe disease (8, 9). Consistently, the current study demonstrates that patients with sHPT exhibited more severe cardiac disease compared to those without, as evidenced by significant differences in NYHA functional class, NT-proBNP levels, and Bethesda classification. Consequently, in Models B and C, advanced heart failure- reflected by elevated NT-proBNP levels and higher NYHA functional class- was associated with sHPT in ACHD. Despite the relatively young age of our cohort, sHPT was prevalent and independently associated with reduced renal function, suggesting that disturbances in mineral metabolism may occur early in the disease course. Importantly, sHPT in ACHD appears to be multifactorial and not solely dependent on chronic kidney disease, but also related to cardiac dysfunction, altered hemodynamics, chronic neurohumoral activation, and vitamin D deficiency (8, 9, 34). In a prior study, we demonstrated that Fontan patients with sHPT had a higher incidence of prior hospitalizations for worsening heart failure and atrial arrhythmias (35). Similarly, in non-ACHD populations, sHPT has been associated with increased cardiovascular and all-cause mortality, as well as higher rates of heart failure–related hospitalizations (36–38). Moreover, elevated PTH has been linked to myocardial remodeling, fibrosis, and vascular dysfunction (12, 13).

A total of 54.7% of patients with sHPT had concomitant vitamin D deficiency, whereas 45.3% exhibited sHPT despite normal vitamin D levels, indicating that sHPT in ACHD cannot be explained by vitamin D deficiency alone. Although vitamin D supplementation may reduce PTH levels, normalization may not be achieved in all patients, as additional factors such as impaired renal function, diuretic therapy, impaired cardiac function, neurohumoral activation, and hepatic congestion contribute to PTH elevation in this population. Taken together, PTH may represent a useful marker within a broader risk stratification framework rather than a standalone therapeutic target.

The sHPT group showed marked impairment across multiple organ systems, including reduced GFR, elevated liver function tests, indicating potential liver impairment due to cardiac dysfunction, lower oxygen saturation, and a higher prevalence of metabolic syndrome. Metabolic syndrome is a known risk factor for chronic kidney disease, with sHPT being a common complication of chronic kidney disease (39, 40). ACHD have an increased risk of developing metabolic syndrome (41, 42). Since metabolic syndrome is a significant risk factor for cardiovascular disease, chronic kidney disease, and ultimately sHPT, metabolic syndrome represents a relevant target for prevention and management in ACHD. Furthermore, reduced exercise frequency was independently associated with sHPT in the multivariate analyses. The association with reduced exercise likely reflects physical limitations, which may be imposed by more severe heart disease in this patient group. On the other hand, infrequent exercise and a sedentary lifestyle are well-established risk factors for metabolic syndrome and cardiovascular disease (43, 44).

Additionally, lower QoL and reduced alcohol consumption consistently emerged as significant factors associated with sHPT across all models. QoL also means being part of groups and joining events with family and friends. Thus, it is not surprising that patients without sHPT consumed alcohol more frequently. Among patients without sHPT, the mean alcohol consumption was 1.8 drinks per week, suggesting that their alcohol intake may be driven by shared social dynamics rather than underlying behavioral disorders. Consistently, ACHD patients with higher alcohol consumption exhibited a significantly lower mean UCLA isolation score, better QoL, greater health satisfaction, and a lower functional NYHA class. In this study, 75% of participants rated their overall QoL as good or very good. This result is consistent with findings from the APPROACH-IS study, which also reported a generally positive QoL among ACHD (45).

No differences were observed in anxiety or depression scores (HADS-A and HADS-D) between the sHPT and no-sHPT group and neither depression, anxiety, or both were identified as independent predictors for sHPT. Nevertheless, screening for depression and anxiety disorders relied solely on the HADS, which may have missed some diagnoses. The accuracy of the HADS-D as a screening instrument in ACHD has been described as moderate previously (46).

### Vitamin D deficiency

In summary, patients with vitamin D deficiency were younger, exercised less frequently, and were less likely to follow a vegetarian diet. They also consumed alcohol less often. Digital technology significantly shapes the lifestyle of children and young adults, leading to more indoor leisure activities and increasing the risk of vitamin D deficiency (47). Furthermore, a vegetarian diet demonstrated superior dietary quality compared to overall diet patterns, contributing to improved health outcomes (48). Therefore, vitamin D deficiency may be linked to lower health consciousness in younger individuals.

However, unlike sHPT, no significant differences were observed in Bethesda class and NT-proBNP levels between patients with and without vitamin D deficiency.

Multivariate models highlighted reduced overall QoL and less frequent alcohol consumption as predictors of vitamin D deficiency (Models D and E). Consistently, a study of 1,084 Swedish women found a positive association between moderate alcohol consumption and higher 25(OH) vitamin D levels (49).

Lower exercise frequency and shorter duration of exercise were important contributors to vitamin D deficiency (Models E and F). These results align with established evidence linking physical activity to higher vitamin D levels (50).

Living in a partnership was identified as a protective factor against vitamin D deficiency (Model F), potentially reflecting the positive influence of a partner in promoting healthier behaviors, such as maintaining a balanced diet, engaging in outdoor activities, or adhering to supplementation. Social relationships contribute to better health outcomes through emotional and practical support in general (51).

Prior studies found that depression is linked to vitamin D deficiency (52). However, in ACHD, neither univariate nor multivariate analyses revealed any association between depression or anxiety and vitamin D deficiency or sHPT. Furthermore, the mean HADS-A, HADS-D, and total HADS scores were within the normal range, indicating an absence of depression or anxiety in this population.

Prior studies showed that obesity, hypertension, and diabetes mellitus are associated with lower 25(OH)D levels (53). Our findings revealed a higher prevalence of metabolic syndrome in patients with sHPT and/or vitamin D deficiency, although the association with vitamin D deficiency narrowly missed statistical significance. Notably, while metabolic syndrome emerged as a significant predictor for sHPT in ACHD, it was not identified as a predictor for vitamin D deficiency in either univariate or multivariate regression analyses.

### Overlapping and diverging predictors

While both sHPT and vitamin D deficiency were associated with reduced QoL and less frequent alcohol consumption, the predictors for each condition diverged in other areas.

Metabolic syndrome and elevated NT-proBNP levels were significant indicators of sHPT, but not of vitamin D deficiency, underscoring the role of cardiovascular and metabolic conditions in the former. Conversely, lifestyle factors such as exercise and social factors like living in a partnership were more prominent determinants of vitamin D deficiency.

### Limitations

This study has several limitations. The first limitation of our study is that it was conducted at a single center, which may limit the generalizability of our findings. Nevertheless, our study features a typical ACHD cohort, consistent with those presented in previous ACHD studies. Given the cross-sectional design of this study, the observed associations do not allow conclusions regarding causality or temporal relationships. Furthermore, the reliance on self-reported data for lifestyle and psychosocial factors may introduce bias.

## Conclusion

This study highlights the distinct and overlapping factors associated with sHPT and vitamin D deficiency in ACHD patients. sHPT appears to be closely associated with advanced cardiac disease and heart failure and may serve as a marker of increased disease burden. Accordingly, assessment of sHPT may contribute to a more comprehensive risk stratification in ACHD. Further studies are needed to determine whether targeted interventions improve clinical outcomes.

## Data Availability

The datasets presented in this article are not readily available because the dataset analyzed in this study contains sensitive patient information and cannot be shared publicly due to ethical and legal restrictions imposed by the institutional ethics committee and data protection regulations. Data access may be granted to qualified researchers upon reasonable request and with appropriate institutional approvals. Requests to access the datasets should be directed to loeffler.friederike@mh-hannover.de.

## Glossary

ACHD: adult congenital heart disease
ALT: alanine aminotransferase
AST: aspartate aminotransferase
APRI: Aspartate Aminotransferase to Platelet Ratio Index
BMI: body mass index
CHD: congenital heart disease
CHE: Cholinesterase
CRP: C reactive protein
EF: ejection fraction
GDF-15: growth differentiation factor 15
GFR: glomerular filtration rate
HADS: Hospital Anxiety and Depression Scale
HADS-A: Hospital Anxiety and Depression Scale - Anxiety subscale
HADS-D: Hospital Anxiety and Depression Scale - Depression subscale
HbA1c: glycated hemoglobin A1C
NT-proBNP: N-terminal pro-B-type natriuretic peptide
NYHA: New York Heart Association
QoL: quality of life
PAH-CHD: pulmonary arterial hypertension in congenital heart disease
PTH: parathyroid hormone
RAAS: renin-angiotensin-aldosterone system
sHPT: secondary hyperparathyroidism
SpO2: oxygen saturation
yGT: gamma-glutamyl-transpeptidase
