# Supplementary material for: The impact of psychosocial factors on secondary hyperparathyroidism and vitamin D deficiency in adults with congenital heart disease—the CHD-HYPER study
Source: Front Nutr. 2026 Apr 27;13:1681346. doi: 10.3389/fnut.2026.1681346 (PMC13159197; doi:10.3389/fnut.2026.1681346)
Supplement: Supplementary file 1 [file Table_1.DOCX]

|  | **Model A *(n=469)***  OR (95% CI) | *p value* | **Model A, adjusted *(n=469)***  OR (95% CI) | *p value* | **Model B *(n=642)***  OR (95% CI) | *p value* | **Model B, adjusted *(n=642)***  OR (95% CI) | *p value* | **Model C *(n=455)***  OR (95% CI) | *p value* | **Model C, adjusted *(n=455)***  OR (95% CI) | *p value* |
| --- | --- | --- | --- | --- | --- | --- | --- | --- | --- | --- | --- | --- |
| ^1^Metabolic Syndrome | 2.175 (1.080-4.380) | *0.030* | 1.989 (0.972-4.070) | *0.060* | 1.653 (0.874-3.126) | *0.122* | --- | --- | 2.391 (1.176-4.861) | *0.016* | 2.091 (1.013-4.315) | *0.046* |
| ^2^NYHA-Class | 1.530 (1.027-2.277) | *0.036* | 1.209 (0.781-1.872) | *0.395* | 2.126 (1.520-2.976) | *<0.001* | 1.623 (1.124-2.345) | *0.010* | --- | --- | --- | --- |
| ^3^Bethesda Class | 0.917 (0.619-1.357) | *0.663* | --- | *---* | 1.125 (0.779-1.624) | *0.531* | --- | --- | --- | --- | --- | --- |
| ^4^Quality of Life | 0.569 (0.386-0.839) | *0.004* | 0.594 (0.398-0.886) | *0.011* | 0.775 (0.557-1.078) | *0.130* | --- | --- | 0.596 (0.402-0.883) | *0.010* | 0.646 (0.430-0.969) | *0.035* |
| ^4^Health Satisfaction | 1.154 (0.790-1.686) | *0.457* | --- | *---* | 0.995 (0.707-1.401) | *0.979* | --- | --- | --- | --- | --- | --- |
| ^5^School Education | 0.959 (0.617-1.491) | *0.853* | --- | *---* | 0.902 (0.614-1.325) | *0.598* | --- | --- | --- | --- | --- | --- |
| ^6^Frequency of Exercise | 0.810 (0.596-1.101) | *0.179* | --- | *---* | 0.745 (0.569-0.976) | *0.033* | 0.775 (0.573-0.995) | *0.046* | --- | --- | --- | --- |
| Alcohol Consumption | 0.534 (0.304-0.937) | *0.029* | 0.541 (0.304-0.961) | *0.036* | 0.612 (0.372-1.007) | *0.053* | 0.609 (0.366-1.014) | *0.056* | 0.511 (0.287-0.908) | *0.022* | 0.531 (0.296-0.954) | *0.034* |
| Headache | 0.392 (0.216-0.712) | *0.002* | 0.464 (0.252-0.857) | *0.014* | --- | --- | --- | --- | 0.451 (0.244-0.832) | *0.011* | 0.503 (0.269-0.941) | *0.031* |
| ^7^Total HADS score | --- | --- | --- | --- | 0.995 (0.995-1.037) | *0.815* | --- | --- |  |  |  |  |
| ^8^NT-proBNP | --- | --- | --- | --- | --- | --- | --- | --- | 1.001 (1.000-1.002) | *0.001* | 1.001 (1.000-1.001) | *0.017* |
| Transferrin Saturation | --- | --- | --- | --- | --- | --- | --- | --- | 0.988 (0.963-1.013) | *0.343* | --- | *---* |
| O_2_-Saturation | --- | --- | --- | --- | --- | --- | --- | --- | 0.963 (0.885-1.047) | *0.372* | --- | *---* |
| Gamma-Glutamyl Transferase (gGT) | --- | --- | --- | --- | --- | --- | --- | --- | 1.003 (0.999-1.007) | *0.179* | --- | *---* |
| Age | --- | --- | 1.007 (0.980-1.035) | *0.623* | --- | --- | 1.008 (0.985-1.031) | *0.522* | --- | --- | 1.006 (0.978-1.035) | *0.679* |
| Sex | --- | --- | 0.860 (0.483-1.530) | *0.608* | --- | --- | 1.012 (0.613-1.672) | *0.962* | --- | --- | 0.843 (0.466-1.528) | *0.574* |
| ^9^GFR | --- | --- | 0.976 (0.961-0.992) | *0.004* | --- | --- | 0.973 (0.961-0.986) | *<0.001* | --- | --- | 0.980 (0.964-0.997) | *0.021* |
| ^10^Vitamin D Deficiency | --- | --- | 1.832 (1.025-3.272) | *0.041* | --- | --- | 1.838 (1.094-3.085) | *0.021* | --- | --- | 2.019 (1.113-3.661) | *0.021* |

**Table A1. Detailed Multivariate analysis results for Secondary Hyperparathyroidism.**

Multivariate odds ratios were calculated to assess associations with sHPT. Hyperparathyroidism was defined as a parathyroid hormone level ≥ 65pg/ml. Models A-C were adjusted for age, sex, GFR and Vitamin D deficiency. All models include patients who provided corresponding response in the questionnaire. ^1^defined as the presence of at least three of the following criteria: waist size ≥ 88 cm (female) or ≥102 cm (male), high density lipoproteins < 50 mg/dl (female) or <40 mg/dl (male), blood pressure ≥ 130 mmHg systolic or ≥ 85 mmHg diastolic, HbA1c ≥ 5.7 %, ^2^New York Heart Association Classification, ^3^Complexity of heart defect according to AHA/ACC, ^4^Quality of life and health satisfaction were assessed using a rating scale from 1 to 5, ^5^ Years of Schooling, ^6^Frequency of exercise was assessed using a rating scale from 1 to 4 (never, monthly, weekly, daily), ^7^ Hospital Anxiety and Depression Scale, ^8^N-terminal pro brain natriuretic peptide, ^9^Glomerular Filtration Rate, ^10^Vitamin D deficiency was defined as a 25-Hydroxyvitamin D level <20ng/ml.

|  | **Model D (n=633)**  OR (95% CI) | *p value* | **Model D, adjusted (n=633)**  OR (95% CI) | *p value* | **Model E (n=635)**  OR (95% CI) | *p value* | **Model E, adjusted *(n=635)***  OR (95% CI) | *p value* | **Model F *(n=506)***  OR (95% CI) | *p value* | **Model F, adjusted *(n=497)***  OR (95% CI) | *p value* |
| --- | --- | --- | --- | --- | --- | --- | --- | --- | --- | --- | --- | --- |
| ^1^Metabolic Syndrome | 1.362 (0.829-2.238) | *0.223* | --- | --- | 1.376 (0.838-2.260) | *0.207* | --- | --- | 1.091 (0.599-1.987) | *0.775* | --- | --- |
| ^2^NYHA-Class | 1.087 (0.806-1.466) | *0.583* | --- | --- | 1.023 (0.753-1.389) | *0.885* | --- | --- | 1.023 (0.712-1.469) | *0.903* | --- | --- |
| ^3^Bethesda Class | 1.133 (0.914-1.406) | *0.254* | --- | --- | 1.161 (0.938-1.437) | *0.171* | --- | --- | 1.102 (0.862-1.409) | *0.437* | --- | --- |
| ^4^Quality of Life | 0.882 (0.658-1.026) | *0.083* | 0.784 (0.619-0.992) | *0.043* | 0.833 (0.668-1.040) | *0.106* | --- | --- | 0.889 (0.688-1.148) | *0.367* | --- | --- |
| ^4^Health Satisfaction | 1.003 (0.825-1.293) | *0.778* | --- | --- | --- | --- | --- | --- | --- | --- | --- | --- |
| ^5^Total HADS score | 0.979 (0.952-1.008) | *0.155* | --- | --- | --- | --- | --- | --- | --- | --- | --- | --- |
| ^6^Fast-Food Consumption | 1.506 (1.095-2.072) | *0.012* | 1.370 (0.978-1.919) | *0.067* | 1.494 (1.086-2.053) | *0.014* | 1.363 (0.974-1.908) | *0.071* | 1.240 (0.862-1.784) | *0.246* | --- | --- |
| Meat Consumption | --- | --- | --- | --- | --- | --- | --- | --- | 2.298 (1.118-4.724) | *0.024* | 1.976 (0.905-4.313) | *0.087* |
| Alcohol Consumption | 0.638 (0.458-0.887) | *0.008* | 0.559 (0.393-0.794) | *0.001* | 0.633 (0.456-0.879) | *0.006* | 0.549 (0.388-0.778) | *<0.001* | 0.689 (0.473-1.003) | *0.052* | 0.558 (0.370-0.841) | *0.005* |
| Smoking | --- | --- | --- | --- | --- | --- | --- | --- | 1.035 (0.529-2.026) | *0.920* | --- | --- |
| ^6^Frequency of Exercise | 0.817 (0.677-0.985) | *0.034* | 0.838 (0.689-1.018) | *0.076* | 0.784 (0.652-0.943) | *0.010* | 0.800 (0.660-0.970) | *0.024* | --- | --- | --- | --- |
| ^7^Exercise Duration | --- | --- | --- | --- | --- | --- | --- | --- | 0.999 (0.998-1.000) | *0.004* | 0.999 (0.998-1.000) | *0.005* |
| Living in a Partnership | --- | --- | --- | --- | --- | --- | --- | --- | 0.576 (0.387-0.857) | *0.007* | 0.534 (0.347-0.822) | *0.004* |
| Children | --- | --- | --- | --- | --- | --- | --- | --- | 0.179 (0.760-0.510) | *1.134* | --- | --- |
| Vocational Training | --- | --- | --- | --- | --- | --- | --- | --- | 0.536 (0.315-0.912) | *0.022* | 0.602 (0.335-1.084) | *0.091* |
| Gamma-Glutamyl Transferase (gGT) | --- | --- | --- | --- | 1.002 (0.999-1.005) | *0.295* | --- | --- | --- | --- | --- | --- |
| Transferrin Saturation | --- | --- | --- | --- | 0.994 (0.983-1.006) | *0.345* | --- | --- | --- | --- | --- | --- |
| Age | --- | --- | 0.996 (0.978-1.014) | *0.674* | --- | --- | 0.997 (0.979-1.015) | *0.722* | --- | --- | 1.003 (0.982-1.024) | *0.791* |
| Sex | --- | --- | 1.248 (0.886-1.758) | *0.205* | --- | --- | 1.210 (0.862-1.700) | *0.271* | --- | --- | 1.042 (0.699-1.553) | *0.840* |
| ^8^GFR | --- | --- | 1.011 (1.002-1.021) | *0.017* | --- | --- | 1.010 (1.001-1.020) | *0.026* | --- | --- | 1.081 (0.416-2.082) | *0.889* |
| Vitamin D Substitution | --- | --- | 0.176 (0.103-0.301) | *<0.001* | --- | --- | 0.176 (0.103-0.300) | *<0.001* | --- | --- | 0.158 (0.086-0.291) | *<0.001* |

**Table A2. Detailed Multivariate analysis results for Vitamin D Deficiency.**

Multivariate odds ratios were calculated to assess associations with Vitamin D deficiency. Vitamin D deficiency was defined as a 25-Hydroxyvitamin D level <20ng/ml. Models D-F were adjusted for age, sex, GFR and Vitamin D substitution. All models include patients who provided corresponding response in the questionnaire. ^1^defined as the presence of at least three of the following criteria: waist size ≥ 88 cm (female) or ≥102 cm (male), high density lipoproteins < 50 mg/dl (female) or <40 mg/dl (male), blood pressure ≥ 130 mmHg systolic or ≥ 85 mmHg diastolic, HbA1c ≥ 5.7 %, ^2^New York Heart Association Classification, ^3^Complexity of heart defect according to AHA/ACC, ^4^Quality of life and health satisfaction were assessed using a rating scale from 1 to 5, ^5^Hospital Anxiety and Depression Scale, ^6^Frequency of fast-food consumption and frequency of exercise were assessed using a rating scale from 1 to 4 (never, monthly, weekly, daily), ^7^Exercise duration in minutes per week, ^8^Glomerular Filtration Rate.
